# Effects of bone-conducted vibration stimulation of various frequencies on the vertical vection

**DOI:** 10.1038/s41598-023-42589-x

**Published:** 2023-09-21

**Authors:** Tetsuta Kondo, Yutaro Hirao, Takuji Narumi, Tomohiro Amemiya

**Affiliations:** 1 Graduate School of Information Science and Technology, The University of Tokyo, Tokyo, 1138656 Japan; 2 Information Technology Center, The University of Tokyo, Tokyo, 1138658 Japan; 3 Virtual Reality Educational Research Center, The University of Tokyo, Tokyo, 1138656 Japan

**Keywords:** Human behaviour, Sensory processing, Perception

## Abstract

Illusory self-motion (“vection”) has been used to present a sense of movement in virtual reality (VR) and other similar applications. It is crucial in vection research to present a stronger sense of movement. Bone-conducted vibration (BCV) is a small and generally acceptable method for enhancing the sense of movement in VR. However, its effects on vection have not been extensively studied. Here, we conducted two experiments to investigate the effect of BCV on the vection, which generates an upward sensation under the hypothesis that BCV stimulation to the mastoid processes causes noise in the vestibular system and enhances visually-induced self-motion perception. The experiments focused on the effects of BCV stimuli of different frequencies on the vection experience. The results suggested that 500 Hz BCV was more effective as noise to the vestibular system than other frequency BCVs and improved self-motion sensation. This study examines the effects of BCV with different frequencies on the vection experience and designs a theory for using BCV in VR.

## Introduction

When a visual pattern of motion in a fixed direction is presented to a stationary observer, the observer perceives self-motion in the direction opposite to the visual pattern^1–4^. This sense of self-motion is called vection. One example is the self-motion experienced by a train passenger when an adjacent train starts to move. For a stationary user, this illusion of motion enables the user to experience movement in a more extensive space within a limited real space. It is widely used to virtually present a sense of movement, such as in amusement parks and driving simulators.

Methods to enhance vection have been explored to enable a strong sense of movement.

Presenting visual and non-visual sensory information, such as auditory^5^ and tactile^6–10^ information, enhances vection. This is because vection is a multimodal phenomenon influenced by sensory modalities other than vision. Among the non-visual sensory information, the vestibular sensation is considered particularly important in vection^11,12^, being a sensory modality providing information on body acceleration. Also, vection is reduced by a mismatch between the actual vestibular activity and that expected from vision (i.e., visual-vestibular conflict^13,14^). Several methods have been proposed to enhance the sensation of vection-induced movement by presenting vestibular information consistent with visual images. Typical examples include motion platforms that present motion and vibration synchronized with visual body images^15^ and methods that generate vestibular sensation synchronized with visual images through galvanic vestibular stimulation (GVS) with an electric current^16,17^. Another study reported that GVS directly affects vection induction^18^. However, the motion platform is a large device with limited space for installation. Although small, GVS has problems such as difficulty presenting vertical acceleration and the pain and tactile sensation caused by electrical stimulation^19^, making these methods unsuitable for general use. This study presents a stronger sense of movement by providing noise to the vestibular senses instead of presenting vestibular sensory information consistent with the vision. This decreases the influence of the vestibular senses and relatively increases the influence of vision. Specifically, we enhance the sense of self-motion by stimulating the mastoid processes (behind each ear) with bone-conducted vibration (BCV) during the vection experience.

Since vection is a multimodal phenomenon, the sense of self-motion in vection is a multisensory integration and perception. Recently, it has become empirically evident that a maximum likelihood estimation model^20^ can explain multisensory integration, where sensory information with higher reliability is given more weight and is considered to have a significant influence on the integration results. This study hypothesized that adding noise to the vestibular system in vection could reduce the reliability of vestibular sensation in self-motion perception and enhance the sense of movement by increasing the relative influence of vision. Recent studies of sensory information presentation use methods to control the results of multisensory integration by adding noise to the sensory system. An example is redirected walking (RDW)^21^, a technology that enables the experience of walking in a vast virtual space within a limited real space by manipulating spatial perception using highly reliable visual information. Recently, it has been shown that noise can be added to the vestibular system using noise vestibular electrical stimulation (nGVS) in RDW to enhance the visual effect^22^. The noise added to vision can enhance the auditory effect^23^, and more effective RDW methods have been proposed.

BCV stimulation is an auditory stimulation where sound is transmitted to the inner ear through the skull bones, bypassing the outer and middle ears. BCV would affect the different vestibular organs^24^ depending on the frequency and stimulation site. BCV predominantly stimulates the otolith organs^25,26^ that detect linear accelerations and head tilts. BCV introduces a mechanical vibration that is translated into an electrical signal by the hair cells within these organs, altering the body orientation information sent to the brain. In contrast, air-conducted (AC) stimulation stimulates the saccule but only the more sensitive cells at the striola of the otolith^27^.

Some studies support that BCV stimulation acts as noise to the vestibular system and alters the vection experience. This is the hypothesis on which Weech and Troje’s^28^ experiments are based, where 500 Hz BCV stimulation of the mastoid processes at the onset of vection shortened the vection latency (the time from the onset of visual motion to the onset of self-motion sensation) during rotational vection. The same method reduces motion sickness^29,30^ and improves the perception of walking with head vibration^31^.

However, the only studies investigating BCV effects on vection have examined the effects on vection latency for the rotational vection mentioned above but not for different vection types (cf. linear vection) or vection magnitudes (self-motion sensory intensity). Moreover, the validation was limited to a 500 Hz vibration stimulus, and the effects of other frequencies have not been clarified. Furthermore, although the factors of eye movements affecting vection have been discussed, they have yet to be verified. Therefore, this study set and verified the following three research questions. The meaning of each research question and its contribution are listed below.

RQ1: Does BCV stimulation influence linear vection experience (especially vection magnitude)? Different organs are responsible for the sense of balance corresponding to the perception of acceleration in response to motion. The semicircular canals account for rotational motion, and the otoliths for linear motion. Therefore, it is not certain that the results obtained for rotational vection will be similar for linear vection. In addition, linear motion is as essential as rotation in movement in VR, and its contribution is considered significant. Considering the results of our preliminary experiments and the burden on the participants, we experimented only on vection that produced an upward sensation among the linear vections.
RQ2: Is there a difference in effect depending on the frequency of BCV stimulation? Whether the effect is seen only in a specific frequency band or over a wide frequency range makes a significant difference in the constraints under which the effect can be utilized. If the effect of vection enhancement can be obtained over a wide frequency range, BCV can be used simultaneously as an acoustic device. For example, BCV can transmit background music or environmental sounds consisting of effective frequency bands in time with VR images. By investigating the differences in the effects of different frequencies, we can explore design guidelines for using BCV stimulation to augment the sense of movement in VR.
RQ3: Do eye movements cause the alteration of the vection experience? Eye movements alter vection^32–36^. By confirming that the alteration of vection experience by BCV is not caused by eye movements, we can examine the validity of the hypothesis in this study that “BCV enhances the sense of motion by providing noise to the vestibular system.” If BCV stimulation acts as noise to the vestibular system, this method compensates for the shortcomings of nGVS (pain and tactile sensation caused by electrical stimulation). This is currently used as noise to the vestibular system, and its contribution may be significant.

## Results

This study conducted two psychophysical experiments on the vection during BCV stimulation of the mastoid processes. In both experiments, seated participants were presented with a random dot that seemed to repeat vertical acceleration and deceleration using a head-mounted display (HMD) while simultaneously receiving BCV to the mastoid processes and acoustic stimulation. In Experiment 1, we compared 125 Hz, 250 Hz, 500 Hz, 1000 Hz, and 2000 Hz vibrations (all pure tones) to the no-vibration condition to identify any difference in the vection experience and to identify the effective frequency range. In Experiment 2, the frequency conditions were narrowed down to 250 Hz and 500 Hz, and “AC stimulation at the same frequency (i.e., presentation of auditory information from the tympanic membrane)” was used as a control group to minimize the differences in auditory experience between the conditions and to examine the effect of BCV stimulation on the vection experience. In both experiments, five items were used as evaluation indices to evaluate the vection experience: vection magnitude, discomfort level, vection latency, vection duration, and eye movement.

The experimental conditions are briefly summarized: Experiment 1 had six conditions, with no vibration as the control condition and five conditions with vibration (125 Hz, 250 Hz, 500 Hz, 1000 Hz, and 2000 Hz, all pure tone) as the experimental conditions. Experiment 2 had two frequency conditions (250 Hz and 500 Hz) and two conduction methods (bone-conduction (BC) and AC) for 2 × 2 conditions.

### Experiment 1: vection experience across diverse vibration frequencies

**Figure 1:**
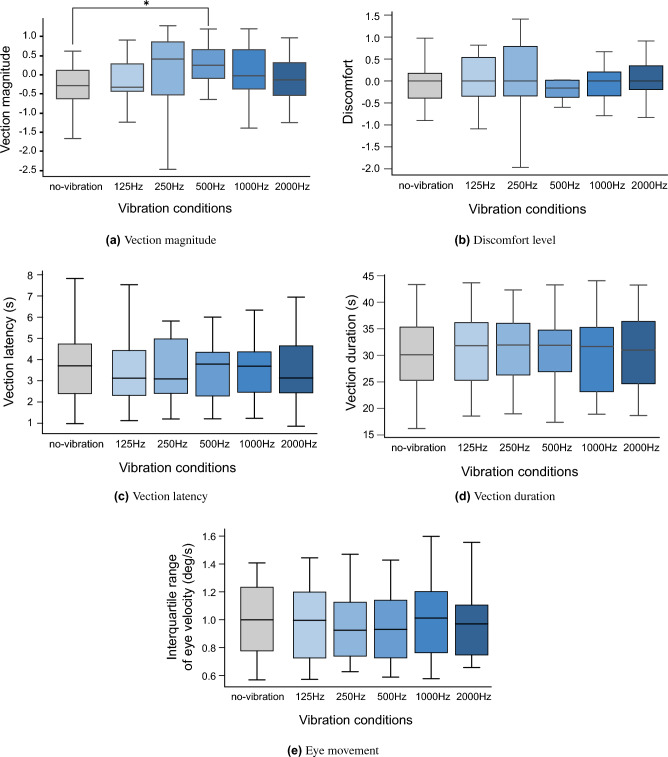
Vertical vection experiences varying frequencies of BCV on the mastoid compared with the no vibration (Experiment 1) (*:$p_{adj}<0.05$).

Figure 1 shows the results of the five indices (vection magnitude, discomfort level, vection latency, vection duration, and eye movement) for the 18 participants, excluding the six participants who were considered to have been strongly affected by the BCV sound. Vection magnitude was − 0.28 ((− 0.62) to 0.12) at no vibration, − 0.32 ((− 0.43) to 0.29) at 125 Hz, 0.41 ((− 0.52) to 0.86) at 250 Hz, 0.25 ((− 0.09) to 0.66) at 500 Hz, − 0.03 ((− 0.37) to 0.66) at 1000 Hz, and − 0.13 ((− 0.54) to 0.32) at 2000 Hz (median (interquartile range)). A significant difference was found between no vibration and 500 Hz ($p_{adj}=0.0384$,$r=0.628$), but not at other frequencies (125 Hz: $p_{adj}>0.05$, $r=0.043$; 250 Hz: $p_{adj}>0.05$, $r= 0.347$; 1000 Hz: $p_{adj}>0.05$, $r=0.315$; 2000 Hz: $p_{adj}>0.05$, $r=0.297$). In contrast, no significant differences in discomfort level, vection latency, vection duration, and eye movement were found between all combinations without and with vibration ($p_{adj}>0.05$).

### Experiment 2: comparing bone-conduction and air-conduction effects on vection experience

**Figure 2:**
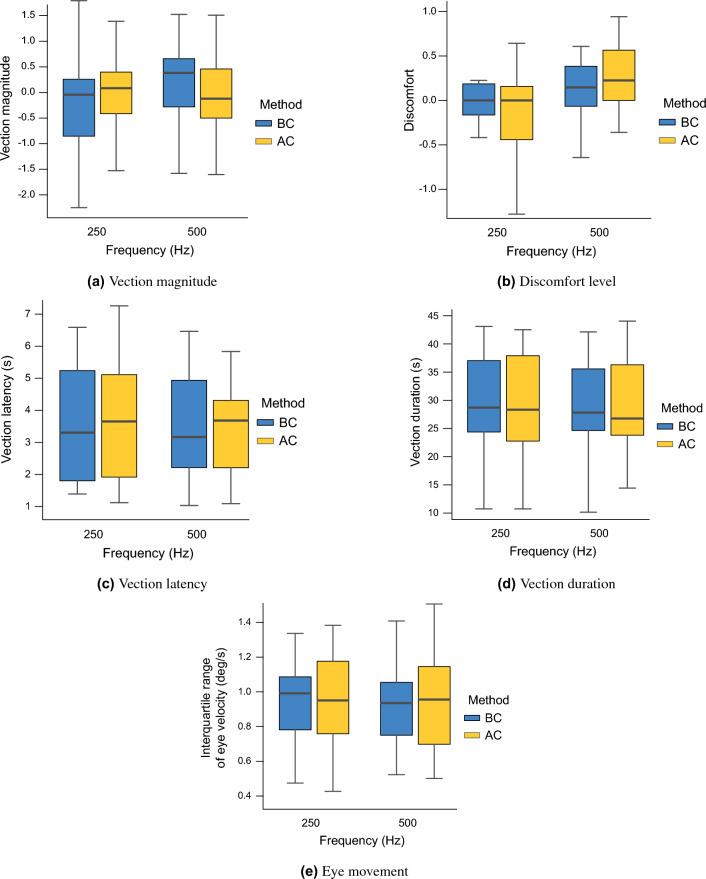
Difference of vertical vection experience between BC and AC sound (Experiment 2).

Figure 2 shows the results of five indices (vection magnitude, discomfort level, vection latency, vection duration, and eye movement) for all 19 participants. After performing an aligned rank transform (ART) on each index, a two-factor analysis of variance was conducted, revealing no significant differences or effect sizes (*p*s $>0.05$) in the main effects or interactions for any indices. This approach decreases the reliability of vestibular sensory information in self-motion perception and increases the reliability of visual information. Therefore, this approach may not be practical for people with low vestibular reliability. In addition, people with low vestibular reliability are expected to have high visual reliability and will be more likely to experience vection. Based on these considerations, we calculated the mean values of the vection magnitude for all trials for each participant, divided them into the “weak-vection group” (10 participants) and “strong-vection group” (9 participants), and performed the same analysis again for each group.

**Figure 3:**
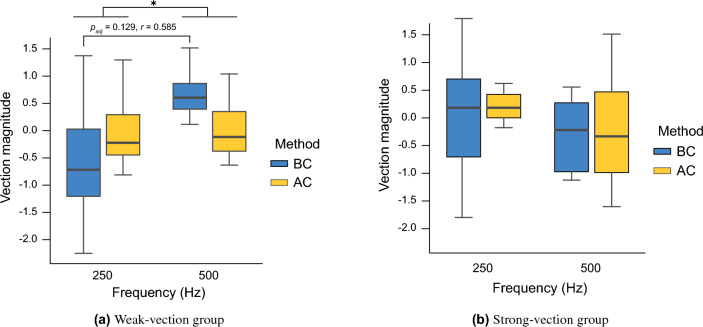
Vection magnitude for each group in Experiment 2 (*:$p_{adj}<0.05$).

Figure 3 shows the results of vection magnitude for each group. Analysis of variance revealed a significant difference (*F*(1,9) = 7.22, *p* = 0.025, partial $\eta ^{2}$ = 0.445) in the frequency main effect in the weak-vection group. However, no significant difference was found for the main effect of the conduction method (*F*(1,9) = 0.105, *p* = 0.75, partial $\eta ^{2}$ = 0.0116). The interaction was also significant (*F*(1,9) = 6.63, *p* = 0.030, partial $\eta ^{2}$ = 0.424). In contrast, in the strong-vection group, no significant differences were found in the main effects and interactions for both frequency and conduction methods (*p*s $>0.05$). Since the analysis of variance for the weak-vection group showed significant differences in the main effect of frequency and interaction, post hoc tests were conducted. The post hoc test results showed no significant differences between any conditions, but a large effect size ($p_{adj} = 0.129$, $r = 0.585$) was found between 250 Hz and 500 Hz in the BC condition.

**Figure 4:**
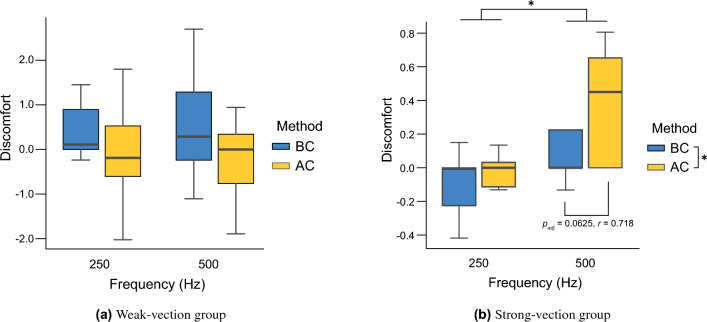
Discomfort level for each group in Experiment 2 (*:$p_{adj}<0.05$).

Figure 4 shows the results of discomfort levels for each group. Analysis of variance revealed no significant differences ($p >0.05$) in the main effects or interactions for either frequency or conduction methods in the weak-vection group. In the strong-vection group, a significant difference was found in the main effect of frequency (*F*(1,8) = 5.46, *p* = 0.048, partial $\eta ^{2}$ = 0.406) and the conduction method (*F*(1,8) = 12.73, *p* = 0.0073, partial $\eta ^{2}$ = 0.614). A significant difference was also observed in the interaction (*F*(1,8) = 8.20, *p* = 0.021, partial $\eta ^{2}$ = 0.506). Because the analysis of variance for the strong-vection group showed significant differences in the main effects of frequency and transmission method and the interaction effects, we performed post hoc tests. The post hoc test results showed a significant effect size ($p_{adj} = 0.0625$, $r = 0.718$) between BC and AC conditions at 500 Hz and no significant difference between BC and AC conditions at 250 Hz ($p_{adj} >0.05$). In addition, no significant difference was found between 250 Hz and 500 Hz for the BC or AC conditions ($p_{adj} >0.05$). The results of the analysis of variance for vection latency, vection duration, and eye movement were not significantly different between BC and AC conditions ($p_{adj} >0.05$).

**Figure 5:**
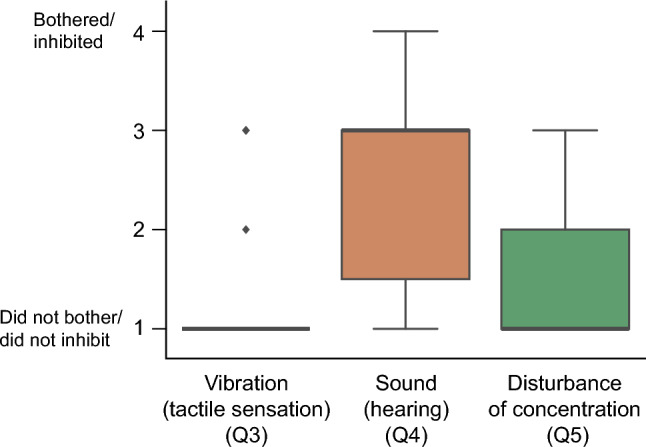
The questionnaire results on the inhibition of attention due to vibration and noise during the vection experience (Q3, Q4, and Q5).

The following are the questionnaire results. Regarding the distinction between BC (i.e., BCV) and AC (i.e., earphones), eight participants answered “yes,” and 11 answered “no” when adjusting the volume (Q1). Six answered “yes,” and 13 answered “no” when experiencing vection (Q2). Figure 5 shows the results of the respondent’s responses to the questions about the inhibition of attention by vibration and sound during the vection experience (Q3, Q4, and Q5). In the open-ended question about whether there was a difference in the vection experience between BC and AC, one participant answered, “It was easier to feel vection with BC.” However, no other responses mentioned a difference in the vection experience. Regarding the difference in loudness between high- and low-pitched sounds, five participants answered that “the high-pitched sound was unpleasant” and “the high-pitched sound was loud.” In contrast, one participant answered that “the low-pitched sound was more unpleasant.”

## Discussion

This study examined the effects of BCV stimulation on the linear vection experience, focusing on the differences in stimulation frequency. Experiment 1 investigated whether there was a difference in the vection experience between the BCV conditions at five frequencies (125 Hz, 250 Hz, 500 Hz, 1000 Hz, and 2000 Hz) and the no-vibration condition. Here, the vection magnitude was stronger only in the 500 Hz BCV condition than in the no-vibration condition. Experiment 2 was limited to the 250–500 Hz range, which significantly affected the vection magnitude in Experiment 1, and an AC condition was set for the control group. It was confirmed that the weak-vection group had a greater vection magnitude at 500 Hz than at 250 Hz. A significant effect occurred between 250 and 500 Hz in the BC condition. These results suggest that 500 Hz BCV stimulation is more likely to enhance the sense of self-motion than other frequencies.

Experiment 2 had no significant difference between the BC and AC conditions at 500 Hz, owing to the smaller sample size as the participants were divided into half groups. In the weak-vection group in Experiment 2, the median vection magnitude of 500 Hz BC was more significant than that of 500 Hz AC. The effect size was moderate ($r = 0.44$), and some respondents answered in the questionnaire that they felt a strong vection with BC. Thus, the possibility that 500 Hz BCV stimulation improves the sense of self-motion is partly suggested by Experiment 2 results. However, these results are insufficient to say that BCV stimulation at 500 Hz improves the sense of self-motion. Additional verification is needed, such as increasing the number of samples or experimenting with images less likely to induce a sense of self-motion. The effect of BCV stimulation on vection magnitude was not observed in the strong-vection group in Experiment 2, suggesting that the effect of this method on enhancing self-motion may be weak for participants who are more sensitive to self-motion. People with low reliability in vestibular sensory information about self-motion sensation are speculated to give more weight visual information about “moving” than vestibular sensory information about “being still” during vection experiences and tend to answer higher vection magnitude^37^. The visual dominance in this study’s method is enhanced by decreasing the reliability of vestibular sensory information. However, the method may be ineffective for people with low vestibular sensory reliability, considering the strong-vection group with low reliability in vestibular sensory information.

There was no difference in the vertical eye movements during the vection experience between the presence and absence of BCV stimulation in both experiments. This suggests that the changes in vection magnitude in the two experiments are unlikely because of eye movements. The results also suggest that the 500 Hz BCV-induced enhancement of self-motion sensation is probably due to BCV stimulation acting as noise to the vestibular system, as hypothesized in this study.

We expected to see a shortening of the vection latency like during rotational vection in the previous study^28^. However, the present experiments showed no difference between the presence and absence of BCV stimulation. No difference in vection latency was also observed in the experiments. The vection latency and vection duration indices are commonly used in vection studies^38,39^ but are unsuitable for our experiments. Visual stimuli in vection studies reach a specific speed when visual motion begins and are presented at that speed for a certain period or until the sense of self-motion disappears. In contrast, our experiments included many acceleration intervals and repeated acceleration/deceleration at a certain period. In this type of visual stimuli, participants could predict the motion to some extent as the experiment progressed, and no difference in vection latency was possible. In addition, most participants lost their self-motion at the end of the deceleration interval. The vection duration almost depended on the vection latency, which did not differ significantly.

Experiment 1 also suggested that BCV stimulation caused differences in auditory stimulation between conditions, significantly affecting the vection experience. Based on our preliminary experiment results, we tried to thoroughly reduce the differences in auditory stimulation by BC sound between conditions in Experiment 1. However, we could not eliminate the BC sound effects on attention and discomfort. In the experimental results, participants who commented that they were “distracted by the sound” or “the sound was unpleasant” tended to report a lower vection magnitude and a higher discomfort level. These results are because of the attenuation of the visual sense of movement in this experiment, as attention was focused on the bone-conducted sound (auditory). The vection experience is considerably affected by presence-related attention^8,40^. In Experiment 2, the control group was placed in an AC condition to control for auditory information between conditions. In the post-experiment questionnaire of Experiment 2, about one-third of the participants could distinguish between BCV and earphones during the vection experience. Most participants answered “1” (did not mind) in the questionnaire about whether the vibration bothered them. These results suggest that the experimental design of Experiment 2 worked well in controlling the experience of the AC and BC conditions and that the participants were unaware of each condition during the experiment. In the questionnaire regarding whether the sound bothered or inhibited attention, most participants answered 2–3 on a 4-point scale for sound awareness and 1–2 for attention inhibition. These results suggest that the volume control was somewhat successful and that the sound did not disturb attention considerably.

Experiment 2 was conducted with both the BC transducers and earphones in position to minimize the effect of a cognitive bias of where sounds originate. This might cause an occlusion effect, where the BC sound is enhanced at low frequencies^41^. The occlusion effect results in a dominance of sound pressure in the ear canal at low frequencies such as 250–500 Hz. Consequently, the sound heard by the participants is mainly from the ear canal sound pressure. However, the purpose of Experiment 2 was to understand whether existence of the mechanical vibration will affect the vection perception by comparing BCV and AC sound. Thus, the conduction path of sound is irrelevant to what we would like to reveal in Experiment 2.

The limitations of this study are as follows. First, it is impossible to eliminate the influence of the sound produced by BCV on vection. Although we minimized the influence of auditory information on vection by setting up a control group in the present experiments, we could not verify the effect of vibration alone because we added vibration at frequencies in the audible range to the mastoid processes. Second, the effect of BCV stimulation as noise on the vestibular system was not sufficiently verified. In this study, BCV stimulation had no significant effect on eye movements during the vection experience, suggesting that the BCV stimulation acted as noise to the vestibular system and thus altered the vection experience. However, it is necessary to conduct another investigation to confirm whether BCV stimulation improves body balance, as confirmed by nGVS^42,43^, and acts as noise to the vestibular system. The third is the validity of the grouping of the participants in Experiment 2. Since this method’s effect might differ from participant to participant and was not assumed prior to the experiment, the grouping was based on the results of the vection magnitude in the experiment. However, it would be more appropriate to divide participants into groups of high and low visual dependence in future experiments by performing a task such as a rod and disk test^44,45^ before the experiment. The fourth is that we did not measure the BC or AC stimulation output in Experiment 2. This made comparing the BCV and AC under the same physical amplitude level challenging since there is no method for physically controlling the output of BC and AC stimulation. Thus, we controlled the stimulation level to minimize the difference in auditory stimulation from the subjective report.

## Methods

### Participants

In Experiment 1, 24 adult participants (10 males and 14 females) aged 20–30 years participated, and the mean age of participants was 24.17 years (standard deviation [SD] = 3.14). After the experiment, the participants were rewarded with an Amazon gift certificate worth 1500 yen (about 11 USD). In Experiment 2, 19 adult participants (10 males and nine females) aged 20–30 years participated, and the mean age of participants was 22.74 years (standard deviation [SD] = 1.52). After the experiment, the participants were rewarded with an Amazon gift certificate worth 1000 yen (about 7 USD). The sample size was approximately twice that of a previous study that investigated the effect of BCV stimulation on vection latency^28^. In both experiments, participant recruitment and experimental procedures were approved by the University of Tokyo ethics committee (approval number: 22-147) and conducted per the principles of the Declaration of Helsinki. All the participants were asked to confirm that they had no problems with vision, hearing, or vestibular function and that they had no aversion to the simulators and provided written informed consent before each experiment. Participants were recruited for each experiment, and Experiments 1 and 2 were conducted on different dates.

### Apparatus

The ALIENWARE m15 R4 gaming laptop (equipped with an Intel Core i9-10980HK processor, NVIDIA GeForce RTX 3080 graphics card, and Windows 10 Pro operation) was used to run the experimental programs. Unity 3D platform (v2019.4.23) was used to develop the experimental program. The visual stimuli in the experiments were presented by an HMD (HTC VIVE Pro Eye; dual OLED displays (3.5 inches) with a resolution of $1440 \times 1600$ pixels per eye ($2880 \times 1600$ pixels total) and a refresh rate of 90 Hz). The HMD is equipped with a 120 Hz eye tracking sensor to measure eye movement during the experiments. In addition, a VIVE controller (with a touchpad and triggers) allowed participants to adjust the sound volume and respond to the self-motion sensory.

**Figure 6:**
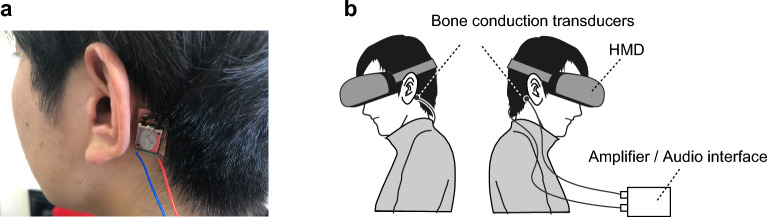
(**a**) Attaching of BC transducer on the mastoid processes. (**b**) Experimental setup.

The experiments used BC transducers (14 mm × 21.5 mm, weight 9.6 g, power handling 1 W RMS/2 W max, impedance 8 $\Omega$), canal-type earphones (ARKARTECH T6), an audio interface (RME/ Fireface UCX), and a digital amplifier (Lepy LP-2024A+) for acoustic stimulus presentation. BC (by BC transducers) and AC stimulations (by earphones) were used for two patterns of acoustic stimulation. The BC transducers were placed on the mastoid processes (behind the ears), as shown in Fig. 6. Kinesio tape was applied to the skin to prevent direct contact between the transducer and the skin, and the transducers were attached to it with double-sided tape. Waveforms of the sound sources were generated in Python (3.10.4) in advance, and the volume was set for each participant before the experiments. The audio interface branched the presentation method (BC or AC). The digital amplifier enhanced the vibration when activating the bone conduction transducers.

### Visual stimuli

**Figure 7:**
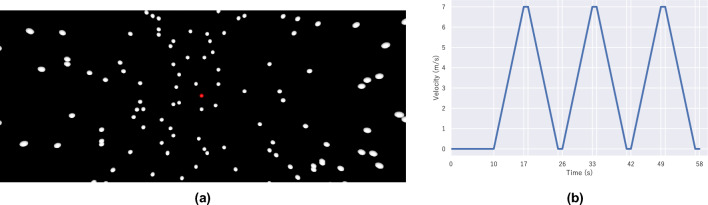
Visual stimuli used in the experiment (**a**: image presented to participants, **b**: transition of movement speed).

The virtual environment used in the experiment was pitch-dark with no ambient light, and 1000 white spheres of radius 0.1 m (approximately four spheres/m^2^) were randomly placed on a cylinder of radius 4 m and height 10 m. A camera and point light source was placed at the center of the cylinder. During the vection experience, all the white spheres moved linearly downward, and the motion was repeated in the cylinder. The participants observed the images from the camera in the virtual environment in the HMD. They perceived upward self-motion, opposite to the sphere’s motion, in the upward direction. In the experiments, a red ball was displayed in the center for a few seconds after the start of the experience to indicate the front (Fig. 7a). Participants were instructed to keep their heads and gaze in the direction of the red ball on display and not significantly move their heads or gaze after it disappeared until the end of the experience. This suppressed the alteration of the vection experience because of eye and body movements during the experience.

In this study’s two experiments, the flow and motion patterns of the vection experience were similar in all cases. Figure 7b shows the visual stimuli velocity. These parameters were adjusted to generate sufficient vection and avoid excessive intoxication. In the experiment, the vection experience was performed in the following sequence using the visual stimuli of the motion pattern described above, and this sequence is called “a single trial.”

1. A white sphere and red dot were displayed in a pitch-black virtual environment, and the user turned their head and gazed toward the red dot.
2. The red dot disappeared 6 s after the image was presented, the white ball began to move 4 s later.
3. The white sphere performs a series of three acceleration/deceleration motions downwards, as shown in Fig. 7b.
4. When the three acceleration/deceleration trials were completed (58 s from the start of the experiment), a questionnaire was displayed in the virtual space, and the participants verbally told their answers to the experimenter.
5. There was a break of approximately 15 s.

BC and AC acoustic stimuli were constantly presented for 58 s from the time the video was presented until the end of the exercise, depending on the condition in the experiment.

### Experimental conditions

#### Experiment 1

Experiment 1 was a six-condition within-participant design, with no vibration as the control condition and five conditions with vibration (125 Hz, 250 Hz, 500 Hz, 1000 Hz, and 2000 Hz, all pure tone) as the experimental conditions. These frequencies were determined with preliminary investigations^46,47^ on vestibular function testing (VEMP) using BCV and our previous studies. In addition to these BCVs, a constant volume of white noise was played through the earphones during the vection experience to block out environmental sounds and noises and to control auditory conditions between the conditions. In the experiment, the order of the six conditions was randomized and repeated three times for 18 trials. The participants determined the volume of each of the five conditions with vibration before the experiment based on the criterion of “the maximum volume that is not unpleasant and allows concentration on the image.” The white noise from the earphones was always played at the pre-determined volume during the volume adjustment and vection experience and could not be adjusted.

#### Experiment 2

Experiment 2 was a within-participant design with two frequency conditions (250 Hz and 500 Hz) and two conduction methods (BC and AC) for 2$\times$2 conditions. In the BC condition, acoustic stimulation was provided by bone conduction transducers attached to the mastoid processes. In the air conduction condition, acoustic stimulation was provided by earphones attached to the ear. To reduce the number of conditions and the participant’s burden, only 250 Hz and 500 Hz frequencies were used, which affected the vection experience based on Experiment 1 results. As in Experiment 1, the participants determined the volume of the four conditions before the experiment based on the criterion of “the maximum volume at which they could concentrate on the images without being uncomfortable.” In particular, to minimize the difference in auditory stimulation between the BC and AC conditions at the same frequency, participants were asked to listen to the conditions alternately and adjust their hearing to sound similar.

### Procedure

Participants first received an explanation of the experimental outline, conditions, and flow and then signed a consent form to participate. The participants sat in a chair, and BC transducers and earphones were placed in each ear. They then adjusted the volume for each acoustic condition used in the experiment. It was explained that the acoustic stimuli of the volume determined here would be played for about 1 min during the vection experience. After adjusting the volume, the participants put on the HMD, held the controller, performed the eye tracking calibration with the VIVE Pro Eye, and began the vection experience. During the vection experience, the participants were instructed to keep pulling the trigger of the controller if they felt any sense of self-motion. After each trial, they were instructed to rate the intensity of the self-motion sensation (vection magnitude) on a scale of 0 to 100 (0: “I am not moving at all, only the ball is moving,” 100: “the ball is stationary and I am completely moving”) and the discomfort on a scale of 0 to 20 (0: “not at all discomforting,” 20: “very discomforting”). They were also instructed to rate the degree of discomfort based on the visual experience alone, without considering the discomfort level of the sound.

In the first part of the experiment, a trial was conducted without any acoustic stimuli to practice manipulation and flow and minimize the effect of habituation on the vection experience on self-motion sensation. Then, three trials were conducted with a break of 1 to 2 min between each set (6 conditions × 3 sets in Experiment 1 and 4 conditions × 3 sets in Experiment 2). After completing all the vection experience trials, the HMD, BC transducers, and earphones were removed. An interview was conducted in Experiment 1, where participants were asked to answer freely about their impressions of the experience orally. A questionnaire was conducted in Experiment 2, where participants answered about the experience, sound, and vibration through a tablet computer (iPad, Apple Inc.). The specific questionnaire conducted in Experiment 2 was as follows.

Q1.: Were you able to distinguish between the BC and earphones when adjusting the volume? Answer “yes” or “no.”
Q2.: Were you able to distinguish between the BC and earphones during your vection experience? Answer “yes” or “no.”
Q3.: Did any vibration (tactile sensation) caused by the BC bother you during the vection experience? Answer on a scale of 1 (did not bother) to 4 (bothered).
Q4.: Did the sound (hearing) bother you during the vection experience? Answer on a scale of 1 (did not bother) to 4 (bothered).
Q5.: Did the sound or vibration inhibit your concentration on the images during the vection experience? Answer on a scale of 1 (did not inhibit) to 4 (inhibited).

In addition to these mandatory questions, the participants were asked to freely answer whether there was a difference in the vection experience between the BC and earphones, whether there was a difference in the experience or loudness between high and low-pitched sounds, and any other comments or observations about the experiment. The time required for the experiments, including breaks, was approximately 60 min in Experiment 1 and 45 min in Experiment 2.

### Data processing

The experimenter recorded participants’ verbal responses to vection magnitude and discomfort level in Excel and eye movement and trigger information during the experience in CSV format at 60 Hz. The data were processed appropriately, and the evaluation values for five evaluation indices (vection magnitude, discomfort level, vection latency, vection duration, and eye movement) were calculated for each trial. The data processing for each index was as follows.

Since there were significant inter-set and inter-individual variations in the vection magnitude and discomfort level, the values obtained for each set were converted into a robust z-score and used as the evaluation value. The robust z-score is a standardized value using the median and interquartile range. It has the advantage of being less affected by data distribution and outliers than standardization using the general mean and standard deviation. A robust z-score is given by: $z = (x_i - x_m) / NIQR$.

Here, $x_i$ represents the value of each data set, and $x_m$ represents the median value. The normalized interquartile range (NIQR) is calculated as $(IQR) \times 0.7413$, where IQR is the difference between the upper and lower quartiles. The robust z-score is with the mean converted to the median and the standard deviation converted to the interquartile range corresponding to the standard normal distribution. For vection latency, the time from the start of the motion until the trigger was pulled was calculated for the three acceleration/deceleration movements in one trial. The average of the three was calculated. For vection duration, the total time the trigger was pulled was calculated for each trial^38,39^. For eye movement, we calculated the average angular velocity of the vertical eye movement every 1/30 s and calculated the interquartile range of the histogram consisting of the angular velocity components^48^ for 48 s from the start to the end of the movement (10 to 58 s after the video presentation). A more extensive interquartile range indicates more significant variability in the velocity distribution, and optokinetic nystagmus (OKN) occurs more frequently. The frames for which no data were available because of blinking or other influences were excluded from the analysis.

### Data analysis

The mean of three trials for each condition was calculated for all evaluation indices calculated for each trial in both experiments and used for the tests. R (Version 4.2.0) was used for all statistical tests.

#### Experiment 1

In Experiment 1, some participants commented in the post-experiment interview that “the sound was unpleasant” and “the sound interfered with my attention.” Experiment 1 investigated the effect of noise to the vestibule by BCV stimulation on the experience of vection, not the effect of sound on the experience. However, the vibration transmitted to the cochlea was perceived as a bone-conducted sound. In addition, it is known that the experience of vection is considerably affected by presence-related attention^8,40^. The experience of vection was likely inhibited by attention to the bone-conducted sound in the present experiment. Therefore, to conduct an analysis that excludes the influence of BC sound thoroughly, data from 18 participants (6 males and 12 females) were used in the analysis, excluding 6 participants whose experiences seemed to be significantly affected by BC sound (comments such as “the sound was unpleasant” and “the sound disturbed my attention” were obtained from the interview results). Wilcoxon signed-rank tests were conducted to examine whether there were differences in the five indices (vection magnitude, discomfort level, vection latency, vection duration, and eye movement) between the presence and absence of BCV stimulation. The significance level was set at 5% and corrected by the Holm method.

#### Experiment 2

Since Experiment 2 consisted of two frequency and two conduction conditions, a two-factor analysis of variance was conducted to examine the main effects of frequency and conduction method and their interaction effects on each of the five indices: vection magnitude, discomfort level, vection latency, vection duration, and eye movement. ARTool^49^ was used to perform a two-factor analysis of variance with a significance level of 5% after ART^49,50^ was performed. If the analysis of variance showed a significant difference in the main effect or interaction, post hoc tests were conducted. In post hoc tests, Wilcoxon signed-rank tests were conducted for the 250 Hz and 500 Hz conditions by conduction method and for the BC and AC conditions by frequency. The significance level was set at 5% and corrected using the Bonferroni method.

## Data Availability

The data is available from T.A. upon reasonable request.
